# The effect of oral nutrition supplement (ONS) on the nutritional and clinical status of patients undergoing autologous hematopoietic stem cell transplantation: study protocol for a randomized controlled clinical trial

**DOI:** 10.1186/s40795-024-00893-3

**Published:** 2024-06-10

**Authors:** Sajedeh Habibi, Seyed Mojtaba Ghoreishy, Hossein Imani, Maryam Barkhordar, Mohammad Vaezi, Erfan Sadeghi, Hamed Mohammadi

**Affiliations:** 1 Department of Clinical Nutrition, School of Nutritional Sciences and Dietetics, Tehran University of Medical Sciences, P.O. Box 14155-6117, Tehran, Iran; 2 Department of Nutrition, School of Public Health, Iran University of Medical Sciences, Tehran, Iran; 3 Student research committee, School of Public Health, Iran University of Medical Sciences, Tehran, Iran; 4 Cell Therapy and Hematopoietic Stem Cell Transplantation Research Center, Tehran University of Medical Sciences, Tehran, Iran; 5 Hematology, Oncology and Stem Cell Transplantation Research Center, Tehran University of Medical Sciences, Tehran, Iran; 6 Department of Biostatistics, School of Medicine, Shiraz University of Medical Sciences, Shiraz, Iran; 7 Research Institute for Oncology, Hematology and Cell Therapy, Tehran University of Medical Sciences, Tehran, Iran

**Keywords:** Oral nutritional supplement, Stem cell transplantation, Clinical trial

## Abstract

**Introduction:**

Several side effects within the patients undergoing hematopoietic stem cell transplantation (HSCT), especially ones that influence nutrition intake, can cause weight loss and malnutrition. Based on studies, oral nutritional supplement (ONS) may reinforce their nutrient intake and progress clinical outcomes. The objective of this research is to investigate the effect of oral nutrition supplements on the nutritional and clinical status of patients undergoing autologous hematopoietic stem cell transplantation.

**Methods:**

After block randomization used the website www.randomization, 38 patients will be enrolled in this study, patients will be allocated to the intervention (ONS) or control groups in a 1: 1 ratio. Patients in the ONS group will receive 250 ml of standard formula (Ensure®, Abbott Nutrition) which has 14–15% protein twice a day, in the morning and bedtime snacks for 21 days. All the procedures done in the control group will be the same as the ONS group except receiving ONS. We will examine the outcomes include; weight, appetite, hand grip strength, calf circumference, mid-arm circumference, total energy intake, protein intake, carbohydrate intake, fat intake, severity of oral mucositis, rate of infection during hospitalization, graft failure, recurrence rate after transplantation, the number of days it takes for neutrophil and platelet engraftment to occur, number of readmissions after transplantation during three months, mortality rate up to three months after transplantation and the three-day food diary record; all the evaluations will be carried out in three steps; 7 days before transplant, on the 14th day after transplantation, and on the 90th day after the transplantation.

**Discussion:**

These patients’ weight loss and malnourishment are significant concerns. The use of ONS in patients receiving HSCT has not been the subject of any research.

**Trial registration:**

This clinical trial was registered in Iranian Registry of Clinical Trials (http://www.irct.ir) on 2022-12-09 with the code number IRCT20220208053971N2.

## Key Points

**Limitations:**

1. Problems related to the possibility of non-cooperation of the hospital and patients.

2. Limitations on available ONS choices and costs.

**Strengths:**

1. The strength of this protocol is the RCT design, as the field of nutrition is limited by a lack of RCTs.

2. Our study is the first RCT on ONS in patients receiving a HSCT.

## Background

Hematologic malignancies can be successfully treated using bone marrow transplantation (BMT) or hematopoietic stem cell transplantation (HSCT) [1, 2]. The procedure involves administering hematopoietic stem cells to a patient through an intravenous infusion after a brief period of chemotherapy and/or radiotherapy, which is called the conditioning regimen [3]. The conditioning regimen’s purpose is to eradicate the underlying malignancy by ablating the recipient’s bone marrow and induce immunosuppression to enable the engraftment of infused stem cells [4, 5]. HSCT includes two main types: autologous and allogeneic. The difference lies in the transplant source and the donor-recipient relationship; in the autologous type, the donor of hematopoietic stem cells is the person her/himself [4, 6]. .

The conditioning regimen, steroids, and immune-suppressors use lead to side effects such as anorexia, mucositis with pain, limited gut absorption, nausea, vomiting, dysgeusia, and diarrhea; all of which have a significant impact on dietary intake; therefore, weight loss is common in this condition [7–9]. Chemotherapy-induced nausea and vomiting and oral mucositis are among the most common side effects that can significantly impact nutrition and overall well-being in myeloma and lymphoma patients receiving autologous HSCT. To mitigate these problems, patients undergoing autologous HSCT are often provided with antiemetic medications such as a combination of a serotonin (5-HT3) receptor antagonist (e.g., ondansetron), a neurokinin-1 (NK-1) receptor antagonist (e.g., aprepitant), and dexamethasone to manage nausea and vomiting and are advised to maintain good oral hygiene practices to reduce the severity of mucositis. Additionally, dietary modifications, such as consuming small, frequent meals and opting for bland, soft foods, can help alleviate these side effects and support adequate nutrition during the treatment process [10].

Before the HSCT, patients have different nutritional statuses; they may be well-nourished, malnourished or at risk of malnutrition. HSCT complications in these patients are linked to both obesity (BMI = 30 kg/m2) and malnutrition (BMI < 18.5 kg/m2) [11]. Some investigations have shown that 10–15% of patients are malnourished before transplantation [12], furthermore, there is a certain risk of remarkable weight loss during treatment [13]. Well-nourished patients generally have shorter hospital stays, higher quality of life (QOL), and higher survival rates compared to malnourished patients [14]. Malnutrition has also been linked to delayed recovery after HSCT, increased hospital readmission rates, and higher mortality [7]. Also, some side effects such as diarrhea, mucositis, nausea, and vomiting reported during the conditioning regimen had impressive adverse effects on nutritional status [15].

As per a meta-analysis oral nutritional interventions are efficient at expanding nutritional intake and improving some aspects of QOL in cancer patients who are malnourished or at nutritional risk; yet don’t seem to improve mortality [16]. Based on previous studies, it has been suggested that healthcare providers should think about providing oral nutrition support to people who can swallow safely and are malnourished or at risk of malnutrition to increase their nutritional intake, reducing side effects and enhancing clinical outcomes [17, 18]. A RCT’s findings indicated that hospitalized hematological patients receiving chemotherapy or radiation therapy loose weight during their hospitalization and consume less energy than needed, so, requiring supplementation [19]. ONS is a successful method of nutritional support, which can reinforce the nutrient content of protein, carbohydrates, fats, minerals, and vitamins in food, and provide balanced nutrients to fulfill the body’s need for nutrients [20, 21]. In a study, Yang et al. concluded: “Esophageal cancer patients undergoing radiotherapy can benefit from ONS, which reduces weight loss and improves nutritional status” [17]. Also, in another study that investigated the effects of nutrition intervention with an ONS during chemotherapy in patients with advanced non-squamous non-small cell lung cancer (NSCLC), found that the nutritional supplement improved patients’ QOL and reduced weight loss in the intervention group compared to the control group [22].

So far, no study has investigated the use of ONS in patients undergoing HSCT; on the other hand, the patients’ tolerance as well as the efficacy of ONS in these patients may be affected by GI problems caused by pre-transplant chemotherapy and various post-transplant complications such as infections, malnutrition, etc.; therefore, the objective of this study is to assess how ONS affects the nutritional and clinical status of patients undergoing autologous HSCT. Also, to allow for a more accurate conclusion, the control group will not get ONS.

## Patients and methods

### Participants

This trial will be done in accordance with the guidelines laid down in the Declaration of Helsinki (1964) [23]. The present study will be a randomized controlled clinical trial among patients undergoing autologous hematopoietic stem cell transplantation aged 18–50 years with a body mass index (BMI) < 30 kg/m^2^. The BMT wards of Shariati Hospital in Tehran, Iran, will be the setting for the study. The intervention is planned to begin on December 2022 and end on April 2024.

### Inclusion criteria

In this study, we will recruit (1) patients with lymphoma or multiple myeloma who are candidates for autologous hematopoietic stem cell transplantation; (2) age range 18–50 years; (3) BMI less than 30 kg/m^2^.

### Non-inclusion criteria

Patients will not be included if they are reluctant to cooperate.

### Exclusion criteria

We will exclude patients who receive less than 70% of the ONS of the intervention group. This study has previously been endorsed by the ethics committee of Tehran University of Medical Sciences (IR.TUMS.HORCSCT.REC.1401.017). All patients will read and sign the aware consent.

### Sample size calculation

In this research, in order to compare the changes of the desired outcomes before and after the intervention between the two groups, taking into account the type I error equal to 0.05, the study power of 80% and the effect sizes obtained from similar studies equal to 2.95 and 1.18 based on body weight and PG-SGA (Kim et al. study) [24] and also 1.37 and 1.34 based on body weight and BMI (Torricelli et al. study) [22]. Using G-Power software, the sample size equal to 8, 34, 20 and 20 people was estimated, respectively. Therefore, the final sample size was determined to be 38 people (19 people in each group) based on the largest estimate obtained, i.e., 34 people and also considering the possibility of 10% dropout.

### Study design and intervention

**Fig. 1:**
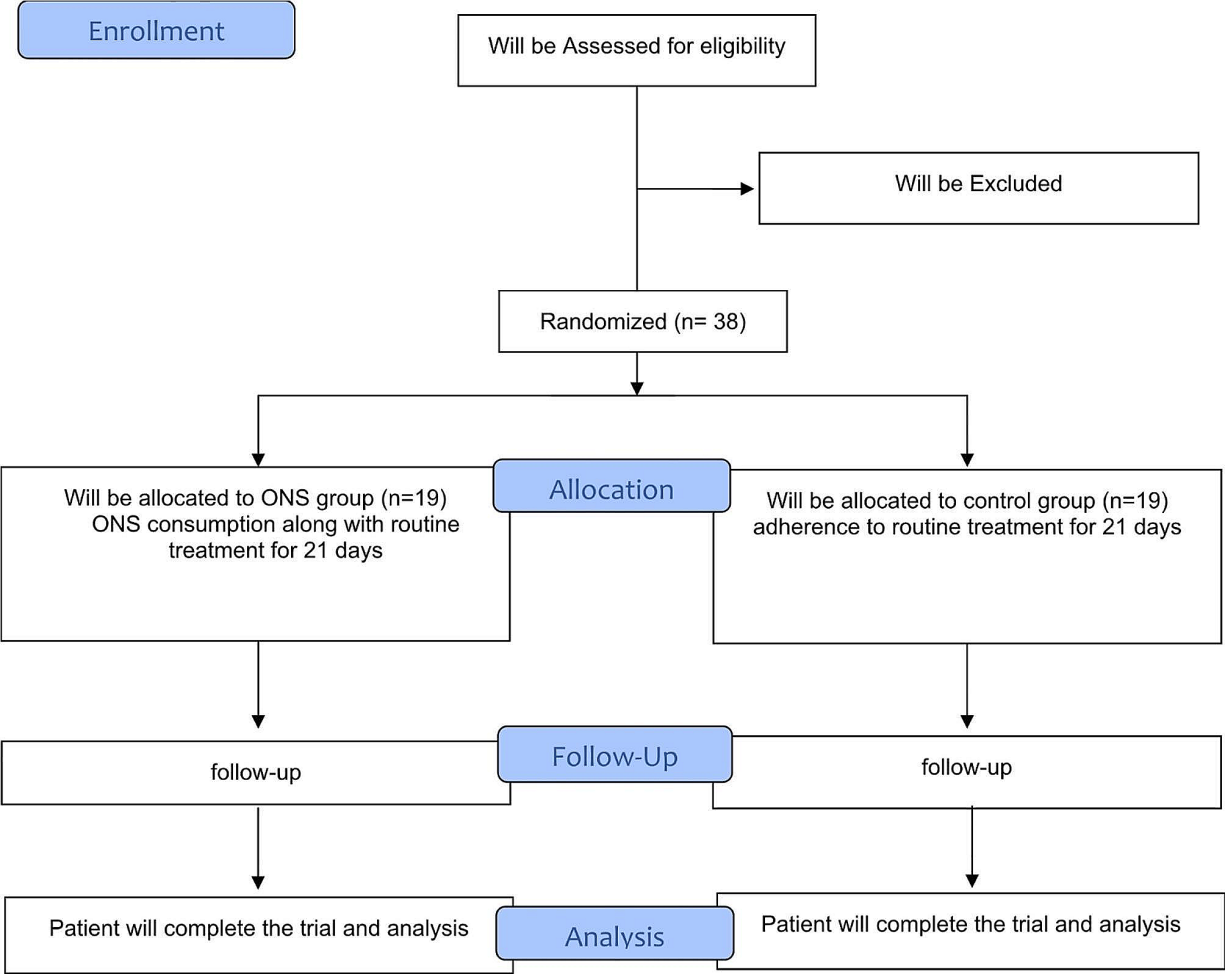
Flow Diagram

Figure 1 illustrates the study design diagram. Inclusion criteria will guide the enrollment process; then patients will be randomly assigned to the intervention (ONS) (*n* = 19) or control groups (*n* = 19). Randomization will be conducted using stratified block design, considering sex and age. The website www.randomization.com will be utilized. The method involves assigning each group either the letter A or B, and then conducting randomization in blocks of size 4. This will be done for sex (male and female) and age (18–35 and 35–50 years old). Within each class, patients will be randomly assigned to one of the two study groups in a 1: 1 ratio. The randomization process will be performed by someone outside the research team.

First, information about age, sex, marital status (single / married), level of education, type of malignancy, past medical history of the disease, tobacco and alcohol use, pre-transplant disease status, pre-transplant disease risk and type of the conditioning regimen will be collected using a face-to-face interview questionnaire. Then, patients in the ONS group will consume 250 ml of standard formula (Ensure®, 1.0 kcal/ml, nutritionally complete and balanced powdered milkshake style in vanilla flavor, Abbott Nutrition) which has 14–15% protein twice a day, in the morning and bedtime snacks (500 ml/ day). Except for not receiving ONS, the procedure of the control group participants will be the same as the intervention group after randomization. Based on past studies and to make a more accurate comparison between the two groups, we will not give the control group any other type of drink in addition to their usual intake [25–29]. Twice a week, patients in both groups will receive personal nutritional counseling during the treatment to consume regular meals as much as possible, especially the protein content of the food, and dietary advice to reduce nausea/vomiting and digestive symptoms. The intervention will be carried out for 21 days and we will follow up with the patients until 90 days after the transplant.

The evaluations will be done in three steps; the first step will be 7 days before transplant, the next step will be on the 14th day after transplantation, and the last evaluation will be on the 90th day after the transplantation. All patients will be in the hospital throughout the intervention and after discharge, only for the third evaluation, 90 days after transplantation, they will come to the outpatient clinic for follow-up. We will also use the three-day food diary to record the patients’ food intake. The outcomes we will examine include; weight, appetite, hand grip strength, calf circumference, mid-arm circumference, total energy intake, protein intake, carbohydrate intake, fat intake, severity of oral mucositis, rate of infection during hospitalization, graft failure, recurrence rate after transplantation, the number of days it takes for neutrophil and platelet engraftment to occur, number of readmissions after transplantation during three months, mortality rate up to three months after transplantation.

In our single center, the conditioning regimen for patients undergoing autologous HSCT is designed to maximize the eradication of malignancies while ensuring patient safety and minimizing toxicity. The regimen is tailored based on the underlying disease, patient age, and comorbidities.

- For multiple myeloma, Melphalan; 200 mg/m² administered intravenously, typically given as a single dose on day − 2 (two days before stem cell infusion).
- For Hodgkin and non-Hodgkin lymphomas. BEAM Regimen: Carmustine (BCNU): 300–600 mg/m² administered intravenously on day − 6.
- Etoposide: 100–200 mg/m²/day from day − 5 to day − 2.
- Cytarabine: 200 mg/m² twice daily from day − 5 to day − 2.
- Melphalan: 140 mg/m² on day − 1.
- To manage nausea and vomiting, a combination of 5-HT3 receptor antagonists (e.g., ondansetron) and NK1 receptor antagonists (e.g., aprepitant) is used.
- Granulocyte colony-stimulating factor (G-CSF) is administered post-transplant to promote neutrophil recovery.
- Broad-spectrum antibiotics, antifungals, and antivirals are provided to prevent infections.

### Compliance to treatment

Throughout the intervention, patients in the ONS group will be given a checklist and asked to write down the exact amount of formula that they consume and also the cause of not consumption, every day by date. They will receive daily reminders via text or call to promote compliance and avoid forgetting to use ONS. They will also get another can of formula if they have consumed the last one. Also, Compliance evaluation will be done by checking the empty cans of formula after consumption and visiting the patient at certain intervals.

### Outcome measures

The primary study outcomes would be weight and BMI. The study’s secondary outcome variables would be the rate of infection during hospitalization, the recurrence rate after transplantation, the severity of oral mucositis, the number of readmissions after transplantation during three months, the number of days it takes for neutrophil engraftment to occur, the number of days it takes for platelet engraftment to occur, graft Failure, mortality rate up to three months after transplantation, appetite, hand grip strength, mid-arm circumference, calf circumference, total energy intake, protein intake, carbohydrate intake, and fat intake.

#### Assessment of food intake

We will use the three-day food diary to record the patients’ food intake (energy, macronutrients and micronutrients) in three evaluation steps; 7 days before transplant, on the 14th day after transplantation, and on the 90th day after the transplantation. Then the recorded values of each food will be converted to grams using the home scales guide and analyzed using Nutritionist IV software.

#### Anthropometric measures

Anthropometric measurements will also be collected at the three mentioned time points; 7 days before transplant, on the 14th day after transplantation, and on the 90th day after the transplantation. Digital scales (Seca, Hamburg, Germany) will be used to measure body weight in a fasting state, without shoes, with minimal clothing, to the nearest 100 g. Standing height will be measured using a tape measure mounted on the wall without shoes with an accuracy of 0.5 cm. BMI will be calculated using the measured height and weight (weight [kg] / height [m^2^]) [30]. To measure the calf circumference in a person who can be standing or sitting on the edge of a bed or chair, place the tape measure around the widest part of the leg and note the size, then take two more measurements in two areas a little higher and lower than the previous location, to be sure of our first measurement. The measurement of mid-arm circumference is taken by placing a tape measure between the shoulder bone and elbow tip with a relaxed hand. Anthropometric measures in patients with edema can be assessed using various methods [31, 32], which we consider in these cases.

#### Assessment of malnutrition based on PG-SGA criteria

To evaluate the nutritional status, we will use the standard PG-SGA tool, which be filled in three evaluation time points by the researcher. The PG-SGA score is a numerical score that represents a global ranking of people with good, moderate, suspected, or severe malnutrition. It includes questions regarding body weight (scores 0–5), food intake (scores 0–4), symptoms affecting food intake (scores 0–23), and physical activity level. An overall score is then created indicating the degree of nutritional intervention required. A score of 9 or more indicates a fundamental need for nutritional intervention [33]. It will be assessed in three mentioned time points; first, 7 days before transplant, then, on the 14th day after transplantation, and the last evaluation will be on the 90th day after the transplantation.

#### Mucositis

We will use the WHO criteria for oral toxicity in cancer treatments to evaluate mucositis. This criterion consists of 5 grades, which we will describe [34].

- Grade 0: no symptoms.
- Grade 1: pain and erythema.
- Grade 2: erythema and ulcers but ability to eat solid food.
- Grade 3: ulcer and can only use a liquid diet.
- Grade 4: oral feeding is not possible.
- It will be assessed in three mentioned time points; first, 7 days before transplant, then, on the 14th day after transplantation, and the last evaluation will be on the 90th day after the transplantation.

#### Infection assessment

Body temperature exceeds 38.3 °C at one time or exceeds 38 °C twice within 1 h. Exist of fever and neutropenia or neutropenia and evidence of infection without fever in a patient, should be confirm by the researcher with an intensive care unit or a consultant infectious disease specialist, and then recorded. Due to the spread of COVID-19, patients will be excluded from the study if they would have a positive PCR for COVID-19 after developing a fever. We will record the infection rate in three steps; the first step will be 7 days before transplant, the next step will be on the 14th day after transplantation, and the last evaluation will be on the 90th day after the transplantation.

#### Neutrophils and platelets engraftment

The first day of 3 consecutive days with an ANC^1^ > 0.5 × 10^9^/L was considered as neutrophil engraftment, while platelet engraftment was defined as the first day of 3 consecutive days with a platelet count > 20 × 10^9^/L [35].

#### Hand grip strength

Right and left hand muscle strength will be measured in a sitting position by holding the hand dynamometer device by the patient, while the patient’s hand is at a 90-degree angle with the elbow without any support; finally, the mean of three trials of grip strength for each hand will be recorded [36]. This measurement also will be done in three steps; the first step will be 7 days before transplant, the next step will be on the 14th day after transplantation, and the last evaluation will be on the 90th day after the transplantation.

#### Appetite

We will use a visual analog scale to assess appetite. This scale consists of 6 questions ranging from 0 to 10 to measure hunger, satiety, desire to eat and a person’s desire for salty, sweet and fatty foods. Appetite status will be measured in three mentioned time points; first, 7 days before transplant, then, on the 14th day after transplantation, and the last evaluation will be on the 90th day after the transplantation.

#### Other variables

After the transplant, we will follow up the patients for three months. We will record graft failure and mortality rate up to three months after transplantation. We will extract the rate of infection during hospitalization, the recurrence rate after transplantation and the number of readmissions after transplantation during three months by using the patients’ medical records and continuous follow-up and attendance at the transplant center of Shariati Hospital.

### Statistical analysis

SPSS software V.24 will be used for all analyses. To check the normality of the variable distribution, we will first use the Kolmogorov-Smirnov test. If the variable does not follow a normal distribution, a logarithmic transformation will be utilized. Means ± SDs or median (IQR) will be used to report the results, as appropriate. Baseline characteristics of study participants will be reported by two groups. Differences in food and nutrient intake throughout the intervention will be tested using Student’s t-test. The effects of intervention on outcome variables will be assessed by the use of covariance analysis. Besides assessing variable means, we will also evaluate and document changes in outcome variables after the intervention. Furthermore, if there is a need to investigate the effect before and after the intervention in each group, paired t-test or Wilcoxon tests will be used. *P* < 0.05 will be considered as statistically significant.

### Patient and public involvement

The trial design, rationale and planned intervention has been discussed and planned with consumers to ensure acceptability. Patients approached for recruitment will be asked to complete a short questionnaire to provide feedback on their concerns relating to nature of interventions they would accept. Those participating in the trial will also be asked for feedback on the trial intervention, the trial process and any difficulties encountered. Information regarding their attitudes towards the implementation of trial findings if positive will also be sought.

## Discussion

In patients with hematologic malignancies, the conditioning regimen prior to HSCT, as well as the use of steroids and immune-suppressors, can cause a variety of side effects, including anorexia, mucositis, limited gut absorption, nausea, vomiting, dysgeusia, and diarrhea. These side effects can all affect dietary intake and result in weight loss [7–9]. Prior to the HSCT, patients’ nutritional statuses might vary. While many patients have normal nutritional status at diagnosis, there is a chance that significant weight loss will occur throughout therapy [13]. Studies have highlighted that weight loss within the first 100 days post-HSCT influences short-term clinical outcomes and one-year overall survival [37]. Additionally, a tailored nutritional pathway with a specific basal energy expenditure rate has been validated to prevent weight loss in HSCT patients undergoing myeloablative conditioning, with skeletal muscle mass identified as an independent risk factor for reduced 2-year survival [38]. In addition, malnutrition was linked to a slower recovery, greater rates of readmission, and increased death following HSCT [7]. One effective way to support nutrition is through ONS, which can increase the amount of macro and micronutrients in food [20, 21]; Additionally, ONS is a crucial therapeutic approach that encourages consuming a lot of protein and calories [39]. According to a study, ONS lessened nausea and vomiting, which are frequently brought on by chemotherapy and can significantly affect one’s appetite and quality of life [40].

Studies have found that ONS can help esophageal cancer patients undergoing radiotherapy by reducing weight loss and improving nutritional status [17]; improve patients’ QOL and reduce weight loss in patients with NSCLC cancer during chemotherapy, the effects of supplementation treatment were also investigated from a metabolic perspective in this study, that it was possible to find that plasma polyamines, one of the well-known markers of tumor growth, was reduced after the treatment [22]. As hematological patients also have less energy intake than they need because of side effects of their chemo(radio)therapy, weight loss is occurred in their hospitalization time; therefore, nutrition support may be beneficial for them to increase their energy intake [19].

The use of ONS in patients undergoing HSCT has not been studied before; however, because GI side effects from chemotherapy and other post-transplant complications may compromise the effectiveness of ONS in these patients, the current study is being conducted to assess how ONS affects the nutritional and clinical status of patients undergoing autologous HSCT. If the nutritional and clinical status of patients receiving autologous hematopoietic stem cell treatment is significantly affected by ONS consumption, then this strategy of increasing total caloric intake and subsequently increasing patients’ weight can be suggested in order to improve their condition.

## Data Availability

No datasets were generated or analysed during the current study.
